# GRADE-ing the Benefit/Risk Equation in Food Immunotherapy

**DOI:** 10.1007/s11882-019-0862-6

**Published:** 2019-04-25

**Authors:** Bettina Duca, Nandinee Patel, Paul J. Turner

**Affiliations:** 1 Section of Paediatrics (Allergy and Infectious Diseases), Imperial College London, Norfolk Place, London, W2 1PG UK; 2 Discipline of Paediatrics and Child Health, School of Medicine, University of Sydney, Sydney, Australia

**Keywords:** Desensitisation, Food allergy, Oral immunotherapy, Outcomes, Safety

## Abstract

**Purpose of Review:**

We reviewed the existing evidence base to desensitisation for food allergy, applying the Grading of Recommendations, Assessment, Development and Evaluation approach to discuss whether desensitisation is likely to become part of routine treatment for patients with food allergy.

**Recent Findings:**

Desensitisation for food allergy to peanut, egg and cow’s milk is efficacious, but whether such interventions are cost-effective is less clear, due to the issues over a sustained desensitisation effect and the increase in allergic reactions occurring in patients on treatment. Few studies have assessed the change in health-related quality of life associated with treatment, and most have not considered discordance between parent-reported changes in health-related quality of life (HRQL) outcomes compared to those of the patients themselves; none to date have controlled for the improvement in HRQL occurring after initial challenge which will confound outcomes.

**Summary:**

The lack of longer-term safety and cost-effectiveness data, as well as an absence of current consensus in the reporting of patient-relevant outcomes, must be addressed in order to be able to recommend the introduction of desensitisation as a routine treatment in healthcare systems.

Despite the first case of food allergy desensitisation being published in 1908 [1], it has taken almost 100 years for this strategy to begin to be evaluated in large, phase 3 trials, including those with commercial sponsorship. There is ongoing debate as to whether this form of treatment is ready for routine clinical practice [2–4], and yet in some ways, the horse has already bolted, with oral immunotherapy (OIT) being offered routinely in many countries, including USA, UK, Spain, Italy and Israel [5, 6].

In this review, we consider the wider context of food immunotherapy: research has demonstrated clear efficacy for desensitisation, particularly with respect to oral immunotherapy [7••]; however, clinical trials are undertaken with significant resourcing and under optimal conditions, and do not generally reflect real-life circumstances. There is a need to evaluate the actual impact of food immunotherapy, both on the patient, their wider family and others who may be affected (e.g. school staff) in order to assess for unintended consequences (or even harm).

## Reducing the Impact of Food Allergy on Affected Individuals and Their Families

Food allergy is a major public health issue, impacting not only affected individuals and those charged with their care, but also having significant implications for food businesses, educational institutions and healthcare systems. The adverse impact of a food allergy diagnosis is comparable to that seen for other chronic illnesses, such as diabetes [8].

**Fig. 1:**
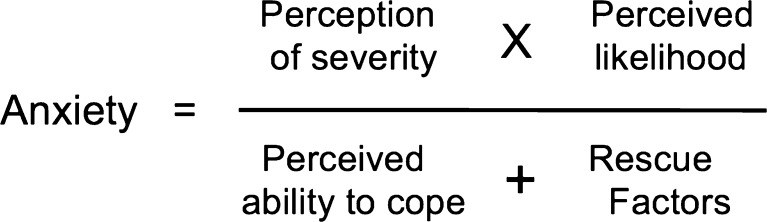
Drivers of anxiety in food allergy and other health conditions. Adapted from [9]. Anxiety is proportional to the perception of danger associated with allergic reactions

The anxiety associated with food allergy can be understood using a similar model to that underpinning cognitive behavioural therapy (Fig. 1), as proposed by Salkoviskis [9]. The drivers of anxiety are the perceived likelihood of a truly life-threatening severe reaction (which, in reality, are very uncommon [10, 11] but also very unpredictable [12]) in combination with a perceived inability to manage severe reactions safely and appropriately.

Food allergy desensitisation will impact these in a variety of ways:

- The majority of clinical trials require an initial screening food challenge: the experience of a controlled reaction under medical supervision can help patients and their families develop a more realistic perception of severity [13, 14].
- In most patients, daily exposure to allergen during treatment will alter their perceived likelihood of reaction. As patients become increasingly desensitised, their *perception* of the likelihood of accidental reactions is likely to improve, despite the fact that patients undergoing desensitisation (at least for oral immunotherapy) tend to experience more allergic events due to breakthrough reactions than patients following allergen avoidance as part of standard care [15••].
- The ability of patients to self-manage any reaction, and their (and their parents’) confidence in doing so, is also likely to improve in studies involving an initial food challenge to confirm allergy, particularly where the subject experiences anaphylaxis which is easily reversed with epinephrine [14].

## The Current Evidence Base

In 2017, the European Academy of Allergy and Clinical Immunology (EAACI) published a systematic review of the current evidence-base for food immunotherapy [7••], which was then used as a basis for a subsequent guideline, using the Appraisal of Guidelines for Research and Evaluation (AGREE II) framework [6]. In this commentary, we have adopted the systematic approach proposed by the Grading of Recommendations, Assessment, Development and Evaluation (GRADE) working group, to evaluate the certainty of findings arising from a systematic review and the challenges in developing recommendations for treatment (Fig. 1) [16–18]. The GRADE framework has been used to develop recommendations for the management of allergic rhinitis [19], as well as the use of probiotics to prevent allergic disease [20].

**Fig. 2:**
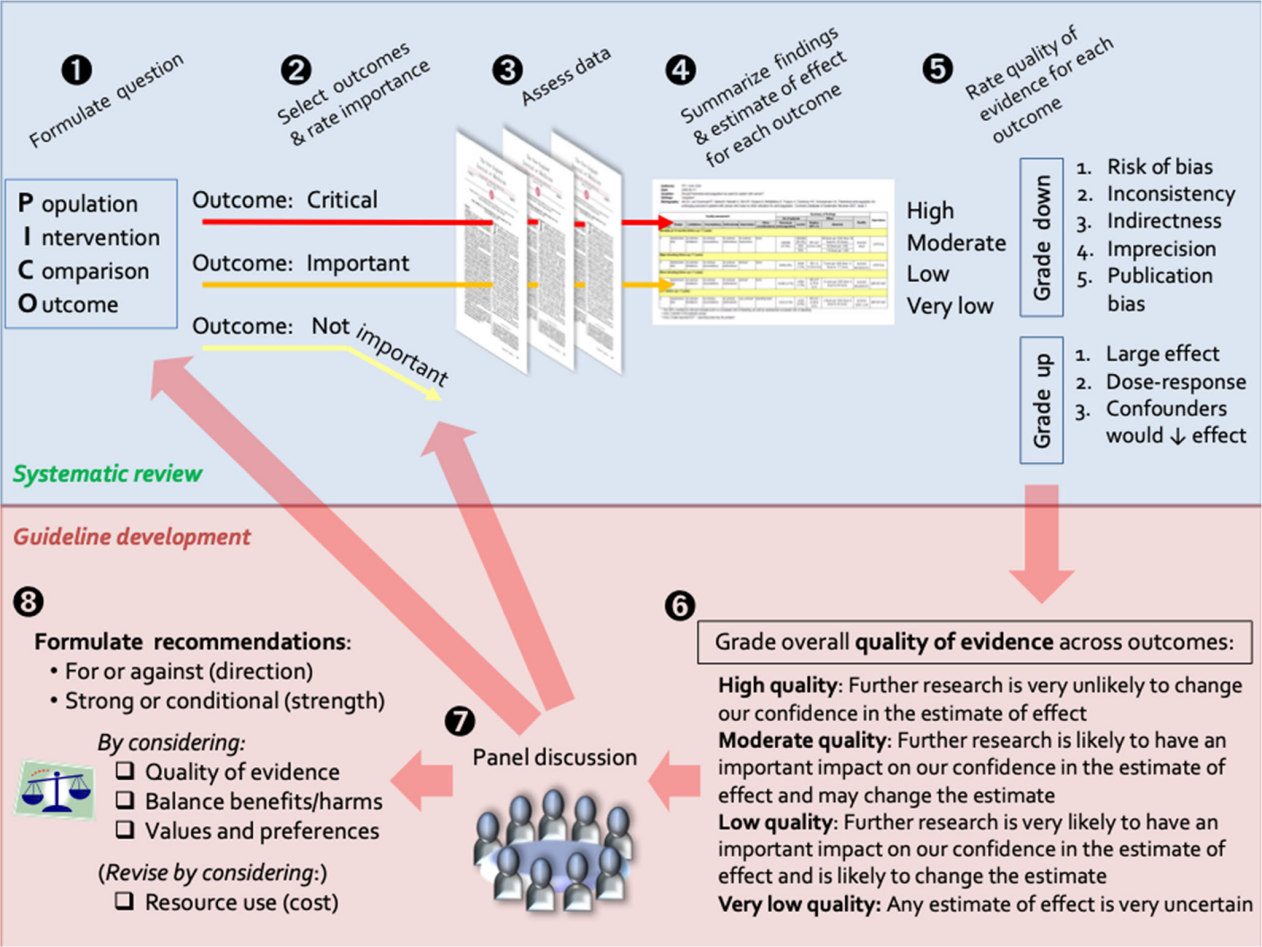
Schematic representation of the GRADE approach for synthesising evidence and developing recommendations. In brief, the available evidence is first assessed for each outcome of interest; quality of evidence may be downgraded for a variety of reasons as listed top right. The applicability of the evidence to the wider population is then evaluated. Figure adapted with permission [21]

In brief, a PICO (population, intervention, comparison, outcome) approach is used to define and evaluate the evidence for each outcome of interest (Fig. 2). Only then is the applicability of the evidence to the wider population assessed. A strong recommendation is appropriate when most patients (or their families) would want the intervention, where the majority of clinicians agree that the intervention should be offered, and where the recommendation is acceptable to policy makers.

## Evaluating the Available Evidence

### Population

There can be significant heterogeneity in participants across studies, due to differences in eligibility criteria. Some studies use a relatively high cut-off for allergen-specific IgE sensitisation as an inclusion criteria (e.g. 7 kUA/l [22•]) compared to others which allow any level of sensitisation. This will impact on both treatment success and safety data: in general, participants who ‘fail’ desensitisation tend to have higher levels of sensitisation and be more prone to treatment-related adverse events (AEs) [4]. Some studies have chosen to exclude patients with a history of anaphylaxis [23] or anaphylaxis (with respiratory symptoms) at baseline challenge [24]. Larger, commercial phase 3 studies may be a more representative of the general food-allergic population; however, they tend to exclude patients who require higher doses of allergen to elicit symptoms. While many phase 3 studies do not exclude patients with prior refractory anaphylaxis (excluding only those with the most severe reactions in the past, for example requiring intensive care), most studies do not report how many participants actually had a history of severe refractory anaphylaxis (as opposed to more typical reactions responding to one or two doses of epinephrine). There is emerging data that younger children may have more successful outcomes with respect to oral immunotherapy, at least for peanut [25]. Thus, the heterogeneity of study populations can cause issues in applying the available evidence to all patient groups.

### Intervention

Correspondingly, there may be significant variation in the intervention(s) used to induce desensitisation. Currently, immunotherapy may be administered via the oral, sublingual or epicutaneous routes [4, 7••], either in isolation or occasionally in combination with each other [26] or other adjuvants such as omalizumab or probiotics [27]. Protocols also vary in terms of allergen form, updosing regimen, maintenance dose and duration. For peanut, most studies have used defatted peanut flour; other studies have used roasted (not defatted) peanut in either ground or solid form [5], while some have used heat-modified peanut to induce desensitisation [28]. The speed of updosing can vary considerably, with some indication that longer updosing periods are associated with improved safety outcomes. Likewise, the dosing level used for maintenance, and its duration of administration, will affect efficacy and safety: studies of peanut OIT have used maintenance doses from 300 to 4000-mg/day peanut protein [29, 30], for durations ranging from a few months [31] to up to 3 years [25]. Lower maintenance doses tend to be associated with more successful outcomes in terms of the proportion of patients achieving a specified level of tolerance; higher maintenance levels may better protect against inadvertent reactions but also result in fewer treatment-related adverse events during updosing [25]—all of which will impact on the risk/benefit equation for patients.

### Comparator: Standard Treatment or Placebo?

Participants undergoing active desensitisation experience more allergic events due to treatment than those following standard care/allergen avoidance [7••, 15••]. Many—but not all—desensitisation studies are undertaken as randomised placebo-controlled trial; such studies tend to report higher rates of AEs than allergen avoidance (standard care) alone. There is no current consensus on whether to correct rates of adverse events for placebo-induced reactions. For example, if a placebo-controlled study reports a 40% rate of gastrointestinal symptoms for subjects on active treatment and 15% for those receiving placebo, is the true rate of symptoms 40% or nearer 25%? Open-label studies risk bias in terms of adverse event reporting, even when incorporating a (non-placebo) control group (e.g. standard care [31] or retrospective controls [25]) and double-blind food challenges are used to assess efficacy outcomes. However, where structured systems are in place to collect AE data, this can lead to an overestimate of adverse events, since the reported rate does not correct for potential placebo-related events.

### Outcomes

The systematic review and meta-analysis undertaken by EAACI in 2017 assessed a number of efficacy outcomes [7••], reporting a range of factors which affected the quality of the available evidence (Table 1). Of note, data was limited with respect to longer-term outcomes and the impact of ceasing regular maintenance dosing. The lack of consistency in the definition and reporting of adverse reactions prevented a meaningful pooled analysis of safety outcomes across studies—something which has been flagged as a major obstacle in using existing data to develop strategies to improve the safety of food allergy desensitisation [4]. The authors had to group ‘desensitisation’ outcomes together, due to the different outcomes used across studies: these vary from achieving a pre-specified clinical threshold or a certain-fold increase in individual threshold at exit food challenge, to a lack of symptoms, to a daily (and typically high) maintenance dose; the heterogeneity in study outcomes has been reviewed by Rodríguez Del Río and colleagues [32••]. The EAACI meta-analysis was further complicated by the use of per-protocol analyses in some studies, rather than intention-to-treat analyses [33, 34].

**Table 1:** Outcome measures assessed by the EAACI systematic review and meta-analysis [7••], with commentary on issues which affect the quality of the evidence

Outcome	Summary of findings	Factors which reduce quality of evidence					Factors which increase quality of evidence
		Study limitations	Inconsistency	Indirectness	Imprecision	Publication bias	
Desensitisation	Meta-analysis demonstrated a substantial benefit in terms of desensitisation (RR = 0.16, 95%CI 0.10, 0.26). Subgroup analyses confirmed both OIT and SLIT are effective. OIT may be less effective in adults.	Failure to conduct intention-to-treat (ITT) analysis in some studies	Heterogeneity across different populations, interventions, outcomes Not all studies utilised DBPCFC to assess outcome. Paucity of data in adults			Fewer, smaller negative studies than expected	Large effect size in children. Some studies suggest a dose-response.
Sustained unresponsiveness	Meta-analysis suggested, but did not confirm sustained unresponsiveness (RR = 0.29, 95%CI 0.08, 1.13)	Failure to conduct ITT analysis in some studies	Heterogeneity across different populations, interventions, definitions used for outcomes Paucity of data in adults			Fewer, smaller negative studies than expected.	
Disease-specific quality of life	Only 1 OIT RCT reported QoL measures: only assessed parental report of QoL and not the participants themselves. No comparison reported between OIT and control group.	QoL only reported in one study, and then by parents and not in patients themselves.		QoL not self-reported by participants	No data in adults.	Only one study	
Safety	Most studies could not be included due to heterogeneity of reporting. *Systemic reactions*: risk higher in those on OIT (RR of *not* experiencing a reaction in controls = 1.16 (95%CI 1.03, 1.30). *Local reactions*: marked increase in the risk (RR of *not* experiencing a reaction in controls 2.12 (95%CI 1.50, 3.0).	Lack of data on long-term adverse outcomes e.g. eosinophilic oesophagitis	Heterogeneity across different populations, interventions, definitions of adverse events used and reporting			Limited data	(Studies without a control group may have bias towards increased reporting of reactions)
Health economic analysis	None of the studies included reported data on cost-effectiveness.	Outcome not assessed	Outcome not assessed		Outcome not assessed	Outcome not assessed	Outcome not assessed

Typically, food challenges are used to determine tolerance to a particular allergen dose; however, the precise definition of ‘tolerance’ is often unclear. Tolerance can vary from a dose being completely tolerated (without any symptoms) to experiencing some lower-grade symptoms so long as they are not ‘dose-limiting’, to more significant symptoms which might be dose-limiting to the patient (e.g. nausea and subjective abdominal pain) but not reach the criteria proposed in the PRACTALL consensus [35]. Unfortunately, there is no current consensus on outcomes—including patient-reported outcome measures (PROMs)—which should be assessed in desensitisation studies.

Importantly, outcomes tend to focus on achieving a certain level of tolerance to a particular dose, but that level is usually defined by the presence or absence of objective symptoms: a patient may therefore experience significant persistent abdominal cramps but this might be deemed ‘tolerated’ according to outcome definitions that require the presence of objective symptoms. Indeed, it is not widely known that the no-observed-adverse-effect level (NOAEL) definition applied to food challenges refers only to objective symptoms: persistent abdominal symptoms in isolation would not impact the NOAEL, despite the fact they are causing the patient to experience an evident and observable adverse event. There is an urgent need to evaluate outcomes which matter to patients and their families, and very much incorporate their views.

Current outcomes may not correspond to patient-desired outcomes (Table 2). Patients may not want to reach a certain level of tolerance, but rather protection from inadvertent allergen exposure. However, there is a lack of evidence for the threshold needed to achieve this, as it will vary between individuals, between allergens, and even within the same individual given the presence or absence of reaction ‘co-factors’ [36, 37]. These issues confound the modelling of the benefits of desensitisation to a population level [38].

**Table 2:** Challenges associated with patient-desired outcome measures for food allergy desensitisation

Outcome measure	Challenges
Reduce risk associated with ‘trace’ and/or more significant accidental exposures	• What is the actual risk associated with traces—do traces cause severe reactions? • What level of desensitisation is protective, given the intra- and inter-person variability in eliciting dose? • Can this level of desensitisation be achieved without ongoing maintenance dosing, which is associated with ongoing risk of adverse events? • Is such a strategy cost-effective?
Desensitisation to allow consumption ad libitum	• This is likely to require ongoing consumption of maintenance doses. • What are the compliance issues (given taste aversion and ongoing low-grade symptoms) associated with long-term maintenance? • Is long term treatment cost-effective?
Longer-term efficacy i.e. tolerance or even ‘cure’	• Is this achievable? • Could we develop predictors of sustained unresponsiveness?
Improve HRQL measures	• What are the drivers of improved HRQL, despite an increase rate of allergic reactions with treatment? • How much does HRQL improve through increased knowledge/awareness/self-efficacy rather than desensitisation?

Only a small number of studies have reported changes in health-related quality of life (HRQL) in participants undergoing food immunotherapy [31, 39, 40•, 41•]; only one [31] was included in the EAACI systematic review. Most studies have used surrogate reports e.g. parent-reported outcomes, for example, using the FAQLQ-PF questionnaire which asks parents to respond on behalf of their child, rather than validated questionnaires completed by the child or young person who is actually undergoing desensitisation themselves, and who may experience low-grade but persisting, treatment-related adverse events [31, 40•, 41]; it is likely that there is discordance between parent-reported FAQLQ-PF and that reported by the child themselves [14]. A notable gap in the literature is with respect to patients who fail desensitisation and the consequential impact on HRQL; in this group of patients, attempts at desensitisation might worsen quality of life (‘my child is too allergic to be treated’).

In summary, current outcomes do not necessarily correspond to outcomes which matter to patients and which therefore should also matter to the healthcare professionals looking after them.

## Translating Evidence to the Real World (See Table 3)

### Priority of the Problem

Despite the adverse impact of food allergy on HRQL [8], treatment of food allergy might not be considered a priority area by healthcare systems. Although food-triggered anaphylaxis is not uncommon, death from anaphylaxis is rare (at approximately 0.03–0.3 deaths/million person years in the general population) [11], and is a rare outcome even amongst the food-allergic population [10]. In consequence, the cost of preventing one death is very high and thus not considered a priority area for healthcare expenditure.

**Table 3:** Applying the evidence to make recommendations

	Patient	Family	Wider population	Health system
Priority of the problem	High due to perception of risk and resulting impact on HRQL		Lower, when considered with life-limiting illnesses such as cancer	Low: fatal food anaphylaxis is a very rare event. Anaphylaxis can be easily controlled through use of rescue medication in the vast majority of cases.
Applicability/generalisability of the evidence	High, where an individual matches the profile of patients included in published trials			
Benefits vs harms	Will vary from patient to patient, depending on the presence of factors which increase the risk of adverse events, issues with compliance and treatment failure. There is currently insufficient data to comment on longer-term benefits/harms. Treatment of a patient may increase risk for other food-allergic members of the household. Some families may opt to undertake their own, unsupervised protocols for other allergens etc.		Lack of understanding in terms of what ‘treated’ food allergy implies e.g. in schools, where patients who have undergone desensitisation may no longer be managed in the same way but remain at risk of unpredictable, severe reactions.	Increased rate of reaction during treatment may increase healthcare costs.
Resource use	Cost (and loss of earnings due to need for frequent visits) may be high, depending on product used and subsidisation by healthcare insurance.		Cost of treatment may not be cost-effective at a population level.	Increased rate of reaction during treatment may increase healthcare costs.
Equity	Significant concerns over cost may limit the treatment to those with sufficient financial income.			
Acceptability	Dependent on occurrence of adverse events for any given patient (difficult to predict) and impact on attendance at school/work	Dependent on family circumstances e.g. need for parents to take time off work for appointments; unintended impact on other food-allergic household members	Likely to be acceptable, but this will depend on whether treatment is funded from a limited pool of resources	
Feasibility	Feasible—and already provided in some geographical regions		There is a need for an international consensus with respect to both outcomes and infrastructure needed to ensure patient safety and allow judgements on cost-effectiveness.	

Nonetheless, there is a clear burden of disease [8]; nut-allergic children have been shown to have poorer overall quality of life as well as emotional, social and psychosocial quality of life compared to data from healthy children [42]. Adolescents with food allergy report that their food allergy had a major impact on their lives, making them feel like they had to be on the alert because of the potential danger of allergic reactions [43]. Food-allergic adolescents and adults have significantly poorer overall health and report limitations in social activities than the general population [44]. Potential improvements in quality of life are used to justify the health-economic benefits of food allergy desensitisation, although to date, it is not clear whether oral immunotherapy for peanut allergy is indeed cost-effective, predominantly due to the uncertainty of longer-term responses and impact of treatment-related and inadvertent reactions [15••].

### Applicability/Generalisability of the Evidence

Over the past decade, the aims of food allergy desensitisation have changed, transitioning from ‘curing’ food allergy to (perhaps) a more realistic target of achieving a sufficient degree of desensitisation to prevent accidental reactions, or at least reduce the risk of severe reactions due to accidental exposures (Table 2). This shift in goalposts, prompted by the reality of a relatively low rate of sustained unresponsiveness reported in the literature, reduces the increase in threshold needed to achieve treatment efficacy. However, as outcomes have shifted to lower, and perhaps more achievable targets, the rates of treatment failure or patient discontinuation have not decreased, correspondingly.

Currently, the strength of evidence for food allergy desensitisation in terms of desensitisation exists only for peanut, cow’s milk and egg [7••]. With the increasing numbers of patients undergoing OIT to these three allergens, in part through large, commercially sponsored phase 3 studies, issues over limited heterogeneity of patients included in OIT trials affecting applicability of evidence have been reduced. Most studies (quite reasonably) continue to exclude those with prior severe reaction requiring intensive care; however, there is often a lack of information over the severity of previous anaphylaxis reactions of included participants, with only a minority of patients experiencing severe anaphylaxis reactions in the past. Few studies have assessed the outcomes in such individuals; those that do have indicated that such patients are less likely to achieve successful outcomes [31]. Arguably, it is these patients who are most likely to benefit from desensitisation, where this can be safely achieved.

### Benefits Vs Harms

The efficacy of food allergy desensitisation, at least for OIT, has been demonstrated [7••]. However, the estimated effect size (such as increase in threshold to elicit symptoms) is more variable, and there are only limited data in terms of the protection offered from inadvertent allergen exposure in the community, which appears to be of primary value to parents [45]. This all comes at a cost: adverse events which appear to be common if frequently self-limiting nonetheless, and a failure rate in around 10–20% of patients. There is a lack of consistency in the reporting of AEs, and significant heterogeneity in AE outcome measures [32••]. Furthermore, breakthrough reactions—including anaphylaxis—do occur in patients established for many months on maintenance dosing [46, 47]—this is likely to be triggered in many cases by the presence of cofactors. Certainly, allergic reactions are ironically more frequent in desensitised patients than in patients following routine allergen avoidance [7••, 15••]. Investigators often claim that the increase in threshold following treatment will protect against ‘trace’ exposures; however, many, if not most, inadvertent reactions involve exposure to non-trace amounts. Furthermore, non-trace quantities which cause reactions may be equivalent to the exposure level of maintenance dosing, which is associated with breakthrough reactions [36, 48].

There have been attempts to model the impact of immunotherapy for food allergy in terms of risk of reaction to unintentional allergen exposure in prepacked foods [38, 49•]. These models are based on exposures to a single food allergen, in patients with allergy to a single food, and would require replication for other allergens; in patients with multiple food allergies, the benefit is less clear. Important assumptions are made with respect to daily exposure risk which may overestimate the reduction in risk expected following treatment. Finally, the models do not incorporate the unintended but likely change in risk-taking behaviours by patients and families following immunotherapy, which may increase exposure risk.

Finally, there is a lack of data relating to longer-term outcomes: the frequency of sustained unresponsiveness where ongoing desensitisation is no longer dependent on regular maintenance dosing, and the freedom this may give to those individuals who wish to consume the allergen ad libitum. A further consideration is understanding how induced desensitisation may differ from natural resolution in terms of longer-term sustained unresponsiveness, and whether desensitisation should therefore only be considered in those unlikely to achieve natural resolution (versus emerging data that younger children may fare better with OIT in terms of desensitisation and adverse events [25]). In addition, thought should be given to other potential unintended consequences, for example, the effect of discontinuation or erratic maintenance dosing due to taste aversion, or impact on other family members such as siblings with food allergy themselves who might be put at increased risk of reactions, a phenomenon that has been reported for the LEAP study (Personal Communication, Prof Gideon Lack).

### Resource Use

There has been minimal work published to date to assess the cost-effectiveness of desensitisation: shaker undertook such an analysis on peanut OIT and concluded while ‘OIT may be cost-effective in a long-term economic model… treated patients may experience a greater rate of peanut-associated allergic reactions and anaphylaxis’ and thus ‘a greater understanding of longer-term risks and benefits is needed before it can be adopted into routine clinical practice’ [15••]. Clearly, the cost-effectiveness of desensitisation would be even more questionable where adjuvants such as omalizumab are included in the protocol, unless this is used to facilitate desensitisation to multiple food allergens concurrently. There is a risk that desensitisation will require increase resource use and might not meet the criteria for cost-effectiveness which are now being applied by organisations such as NICE in the UK. Given the very low incidence of fatal food anaphylaxis [10, 11], it is unlikely that desensitisation will be cost-effective in preventing food allergy mortality: any benefit is likely to be dependent on improved HRQL, as has been the case for venom immunotherapy [50]. Finally, where desensitisation involves essentially the use of food (such as with OIT) rather than a novel product (such as with epicutaneous immunotherapy), health systems will need to consider whether the cost of pharma/GMP-grade food allergen can be justified above the potential to use the food itself to obtain the treatment effect at a fraction of the supply cost [51].

### Equity

The potential cost of desensitisation is likely to result in a lack of equity in terms of access—resulting in desensitisation an option for those families who can afford it—while the majority of allergic individuals would be priced out of the market effectively resulting in a two-tier system and the creation of second-class allergy citizens. Current research protocols are often more geared to achieving success in order to facilitate marketing approval, rather than targeting patients with higher levels of sensitisation, more significant asthma and/or history of more severe anaphylaxis, who may be more prone to treatment-related AEs and yet contribute disproportionately to healthcare costs associated with current standard management and who arguably have most to benefit from desensitisation [4].

### Acceptability

The decision to undertake desensitisation needs to be taken on a per-patient benefit. OIT is associated with high rates of adverse events including anaphylaxis, and thus may not be safe or acceptable to all patients and their families [4]. Issues of adherence can potentially result in life-threatening reactions [52]. Healthcare professionals may not be prepared to take on certain patients as a result. Desensitising younger patients in whom assent cannot be easily assessed presents ethical issues: oral pruritus and abdominal symptoms are common with OIT, but such symptoms can be difficult to communicate at younger ages. Parents and healthcare professionals may not appreciate the presence or impact of non-objective adverse events. In public health systems with limited resources, the cost of desensitisation is likely to be an obstacle, with the cost per life saved far likely to exceed that for other conditions, such as paediatric cancer.

### Is the Intervention Feasible to Implement?

Issues of cost and equity of access aside, it is likely that desensitisation is feasible to implement: OIT is already available routinely from specialist centres in many countries, and in private practice in USA [5]. Crucially, there is a need to obtain longer-term data with respect to these interventions and ensure adequate safety precautions, given the unpredictability of severe reactions. Perhaps the greatest challenge to implementation remains the provision of appropriate training to healthcare professionals. Since desensitisation is not currently accepted as ‘routine care’, training and experience are limited; patient safety must remain the priority, and this can only be achieved through appropriate education for all stakeholders.

Two additional issues need to be considered. The use of food to induce desensitisation, at least with OIT, is a grey area in terms of regulation. In the USA, there are concerns that any food products used as a therapeutic intervention should receive FDA oversight, with the result that practitioners may be forced to use commercially regulated products rather than food. This needs to be considered in the context of the population dose distribution of thresholds: for example, around 50% of peanut-allergic individuals require at least ½ a peanut (100 mg) to trigger objective symptoms. It would seem counterintuitive and even unnecessary to require such individuals to consume milligramme quantities of an expensive pharmaceutical product when cheaper, pragmatic food-based alternatives exist.

Finally, it is essential for an international consensus to be achieved with respect to both outcomes and infrastructure needed to ensure patient safety. Severe food reactions are unpredictable: the occurrence of a just a single death due to desensitisation would severely impact on both clinical availability of the treatment and future research, something which occurred historically in the UK with respect to injected allergen immunotherapy causing at least 26 deaths between 1957 and 1986 which subsequently setback the provision of allergen immunotherapy in the UK for over a decade [53]. More recently, the tragic death of a patient undergoing a routine food challenge in the USA resulted in a press release re-iterating and emphasising the need for appropriate safety precautions and oversight of what is considered to be a routine and generally safe diagnostic test [54]. Such events must serve as a reminder to healthcare professionals undertaking desensitisation in any setting to ensure adequate governance and safety provision for their patients.

## Summary

For healthcare professionals providing care to food-allergic patients, we are working in exciting times. We are now able to offer a treatment for food allergy with the potential to modify the long-term trajectory for these patients, rather than just management strategies revolving around allergen avoidance. However, there is a lack of longer-term safety and cost-effectiveness data for food allergy desensitisation. It is therefore imperative that the profession undertakes further collaborative, consensus-driven research to address these gaps in knowledge and move towards targeted, personalised treatments which do not compromise patient safety or access to care.
